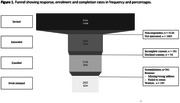# Feasibility and acceptability of APOE genotyping in Dutch Brain Research Registry participants

**DOI:** 10.1002/alz.091894

**Published:** 2025-01-09

**Authors:** Lisa Waterink, Sven J van der Lee, Daphne Nijland, Leonie N.C. Visser, Yolande A.L. Pijnenburg, Sietske A.M Sikkes, Wiesje M. van der Flier, Marissa D. Zwan

**Affiliations:** ^1^ Amsterdam Neuroscience, Neurodegeneration, Amsterdam Netherlands; ^2^ Alzheimer Center Amsterdam, Neurology, Vrije Universiteit Amsterdam, Amsterdam UMC location VUmc, Amsterdam Netherlands; ^3^ Genomics of Neurodegenerative Diseases and Aging, Human Genetics, Vrije Universiteit Amsterdam, Amsterdam UMC, Amsterdam Netherlands; ^4^ Medical Psychology, Amsterdam UMC location AMC, University of Amsterdam, Amsterdam Netherlands; ^5^ Amsterdam Public Health, Quality of Care, Amsterdam Netherlands; ^6^ Division of Clinical Geriatrics, Center for Alzheimer Research, Department of Neurobiology, Care Sciences and Society, Karolinska Institutet, Stockholm Sweden; ^7^ Alzheimer Center Amsterdam, Neurology, Vrije Universiteit Amsterdam, Amsterdam UMC, Amsterdam Netherlands; ^8^ Amsterdam Neuroscience, Neurodegeneration, Amsterdam, Noord‐Holland Netherlands; ^9^ Faculty of Behavioural and Movement Sciences, Clinical Developmental Psychology & Clinical Neuropsychology, Vrije Universiteit Amsterdam, Amsterdam Netherlands; ^10^ Epidemiology and Data Science, Vrije Universiteit Amsterdam, Amsterdam UMC location VUmc, Amsterdam, Noord‐Holland Netherlands

## Abstract

**Background:**

Participant recruitment for Alzheimer’s disease (AD) research and clinical trials is challenging. Online registries can be used to identify and pre‐screen eligible participants. Additional APOE genotyping has the potential to better identify individuals at‐risk for AD. Our goal was to assess feasibility and acceptability of APOE genotyping in cognitively‐normal, older registrants of Dutch Brain Research Registry (DBRR).

**Methods:**

9,511 registrants over the age of 50 with no self‐reported diagnosis of mild cognitive impairment or dementia were invited for APOE genotyping. Registrants were informed that they would not receive their results, yet that results may be used to pre‐screen participants for future studies for which it might be relevant to learn their genetic risk. After providing consent on an elaborate online informed consent procedure including a video, participants were enrolled, and received a buccal swab at home. Feasibility was measured in response rate (%interested/invited), enrolment rate (%enrolled/interested), and completion rate (%returned swabs/enrolled). To assess acceptability, participants answered online questions about our information provision, the project’s aim, and motivation for study participation. APOE genotyping is currently ongoing.

**Result:**

3,393 registrants were interested (35% response rate), of which 3,158 actually enrolled (93% enrolment rate; 57% female; median age [IQR] = 68 [63, 73]), and 2,602 successfully returned the buccal swab (82% completion rate; Figure 1). 20% of enrolled registrants report having subjective memory complaints, and 45% had a first‐degree relative with dementia. Of 1,065 registrants that declined participation, 344 registrants provided reasons which were mostly time related (*n* = 104;30%), health‐issues (*n* = 61;18%), or wanting to receive results of APOE genotyping (*n* = 59;17%). Only 8% (n = 26) replied not wanting to learn their genetic risk. Among the enrolled registrants 2,093 filled out the survey, of which the vast majority was content with our information provision (87‐97%), and had good comprehension about the project’s aim (91‐98%). Most endorsed reasons to participate was ‘to contribute to scientific research’ (97%) and ‘to participate in future research to learn their genetic risk of dementia’ (34%).

**Conclusion:**

Our results suggest that APOE genotyping within an online research registry is feasible and well received for pre‐screening to accelerate inclusion for preclinical AD trials.